# Exonic duplication of the *OTC* gene by a complex rearrangement that likely occurred via a replication-based mechanism: a case report

**DOI:** 10.1186/s12881-018-0733-3

**Published:** 2018-12-12

**Authors:** Katsuyuki Yokoi, Yoko Nakajima, Hidehito Inagaki, Makiko Tsutsumi, Tetsuya Ito, Hiroki Kurahashi

**Affiliations:** 1 Department of Pediatrics, Fujita Health University School of Medicine, Toyoake, Japan; 2 Division of Molecular Genetics, Institute for Comprehensive Medical Science, Fujita Health University, 1-98 Dengakugakubo, Kutsukake-cho, Toyoake, Aichi 470-1192 Japan

**Keywords:** Ornithine transcarbamylase deficiency, Exonic duplication, Complex rearrangement, Fork stalling and template switching (FoSTeS), Non-homologous end joining (NHEJ)

## Abstract

**Background:**

Ornithine transcarbamylase deficiency (OTCD) is an X-linked recessive disorder involving a defect in the urea cycle caused by *OTC* gene mutations. Although a total of 417 disease-causing mutations in *OTC* have been reported, structural abnormalities in this gene are rare. We here describe a female OTCD case caused by an exonic duplication of the *OTC* gene (exons 1–6).

**Case presentation:**

A 23-year-old woman with late-onset OTCD diagnosed by biochemical testing was subjected to subsequent genetic testing. Sanger sequencing revealed no pathogenic mutation throughout the coding exons of the *OTC* gene, but multiplex ligation-dependent probe amplification (MLPA) revealed duplication of exons 1–6. Further genetic analyses revealed an inversion of duplicated exon 1 and a tandem duplication of exons 2–6. Each of the junctions of the inversion harbored a microhomology and non-templated microinsertion, respectively, suggesting a replication-based mechanism. The duplication was also of de novo origin but segregation analysis indicated that it took place in the paternal chromosome.

**Conclusion:**

We report the first OTCD case harboring an exonic duplication in the *OTC* gene. The functional defects caused by this anomaly were determined via structural analysis of its complex rearrangements.

**Electronic supplementary material:**

The online version of this article (10.1186/s12881-018-0733-3) contains supplementary material, which is available to authorized users.

## Background

Ornithine transcarbamylase (OTC) is a mitochondrial urea cycle enzyme that catalyzes the reaction between carbamyl phosphate and ornithine to form citrulline and phosphate [1]. Ornithine transcarbamylase deficiency (OTCD) is one of the most common urea cycle disorders [2] with an estimated prevalence of 1 in 14,000–77,000 [1]. The human *OTC* gene, located on the short arm of the X chromosome (Xp11.4), is 73 kb with10 exons and 1062 bp of coding sequence [3–5]. Because OTCD is inherited in an X-linked manner, deficient hemizygous males usually develop this disorder. However, a remarkable feature of OTCD is that a substantial subset of heterozygous females also develop this condition. The symptoms of carrier females vary in terms of onset and severity. Since the *OTC* gene is subject to X-inactivation, it is believed that this phenotypic variability depends on a skewed degree of this in the livers of carrier females [5].

In 85–90% of patients with a biochemical phenotype of OTCD, a mutation can be identified through sequencing or deletion/duplication testing [6]. A total of 417 disease-causing mutations in the *OTC* gene have been reported to date [1]. Exonic deletions have also been described but no prior case of OTCD caused by exonic duplication has previously been reported [7]. In our current case report, we describe a female patient with OTCD caused by a partial duplication of *OTC* exons 1–6.

## Case presentation

### Patient

The current study patient was a 23-year-old woman with normal psychomotor development and healthy nonconsanguineous parents. She had frequent episodes of nausea, vomiting, stomachache and temporary elevated transaminase from about 4 years of age. Ammonia and plasma amino acid levels were measured when she was 5 years old. Her serum ammonia was 220 *μ*g/dl (normal range 12 ~ 60 *μ*g/dl) and she showed high levels of glutamine (1212 nmol/ml; normal value, 420–700), lower normal limits of citrulline (18.4 nmol/ml; normal value, 17–43), and lower plasma levels of arginine (32.2 nmol/ml; normal value, 54–130). A urine metabolic screen indicated a gross elevation in orotate (orotate/creatinine ratio 234.3 *μ*mol/g creatinine; normal value, 4.7 ~ 15.9 *μ*mol/g creatinine). These findings were consistent with OTC deficiency. She was therefore biochemically diagnosed with OTCD and her blood ammonia level has been well controlled since by a protein-restricted diet and by oral sodium phenylbutyrate and arginine. Recently, we performed genetic analysis to identify the genetic alterations of the *OTC* gene in this patient. However, Sanger sequencing revealed no pathogenic mutation.

### Genetic analysis

#### Mutational analyses

Sanger sequencing was performed to screen for genetic variations at the nucleotide level throughout all coding exons of the *OTC* gene (Additional file 1). We used UCSC genome browser (http://genome-asia.ucsc.edu/) as human genome assembly. To screen for exonic deletions or duplications, multiplex ligation-dependent probe amplification (MLPA) was performed using the SALSA P079-A3 *OTC* MLPA kit (MRC Holland, Amsterdam, The Netherlands), in accordance with the manufacturer’s recommendations. MLPA products were separated by capillary electrophoresis on an ABI3730 genetic analyzer and then processed using GeneMapper software. The peak heights of the samples were compared with control probes and the ratios of these peaks were calculated for all exons. If the dosage quotient was 1.0, the results were considered normal. Thresholds for deletions and duplications were set at 0.5 and 1.5, respectively.

#### Quantitative real time PCR

**Fig. 1:**
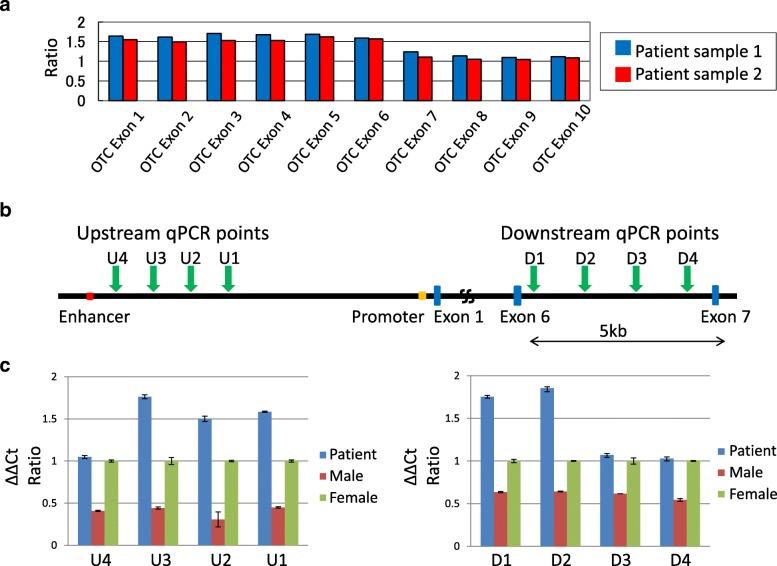
Exonic duplication of the *OTC* gene in a female OTCD patient. **a** MLPA results for the *OTC* gene in this patient indicting duplication of exons 1–6. **b** Structure of the *OTC* gene. The blue boxes denote exons. The enhancer and promoter regions are shown in red and orange, respectively. The positions of the qPCR units are indicated by green arrows. Upstream qPCR units: U1 - U4. Downstream qPCR units: D1 - D4. **c** qPCR results. The left and right panels show the qPCR findings for the upstream and downstream regions, respectively. The blue bars indicate the patient and the red and green bars denote the male and female controls, respectively. The Y- axis indicates the ΔΔ Ct ratio

To demarcate the duplicated region, quantitative real-time PCR was conducted on blood DNA from the patient and a male control subject using the Applied Biosystems 7300 real time PCR system (Thermo Fisher Scientific). Several primer pairs were designed for *OTC* (upstream of exon 1 and intron 6) and *RPP30* that was used as an autosomal single copy gene reference to generate amplicons suitable for real-time PCR (Fig. 1b, Additional file 1). The PCR reaction was performed in a 15 μL reaction system, containing 2 μL of template DNA (5 ng/μL), 0.6 μL of each primer set (10 μmol/L), 0.3 μL ROX Reference Dye, 4 μL distilled water, and 7.5 μL of 2xTB Green Premix Ex TaqII (Tli RNaseH Plus, TaKaRa). Two parallel PCR reactions were prepared for each sample. The amplification cycling conditions were as follows: 95 °C for 30 s, followed by 40 cycles at 95 °C 5 s and 60 °C for 1 min. Data evaluation was carried out using the 7300 system SDS software and Microsoft Excel. The threshold cycle number (Ct) was determined for all PCR reactions and the same threshold and baseline were set for all samples. The starting copy number of the samples was determined using theΔΔCt-Method. ΔΔCt method was a modification of the method described in Livak et al. for quantifying mRNA [8]. ΔCt represents the mean Ct value of each sample and was calculated for *OTC* and *RPP30*. The starting copy number of the unknown samples was determined relative to the known copy number of the control sample using the following formula:

ΔΔCt = [ΔCt OTC(patient)-ΔCt RPP30(patient)] - [ΔCt OTC(female)-ΔCt RPP30(female)]. The relative gene copy number was calculated by the expression2^-Δ(ΔCt)^. The starting copy number of male control was also determined as a reference value.

#### Inverse PCR

Inverse PCR were performed using restriction enzyme *Taq*I (TaKaRa, Shiga, Japan) to isolate the unknown sequences adjacent to the duplicated region of the *OTC* gene in the study patient. ApE – A plasmid Editor software was used to identify the recognition sites for the restriction enzyme. The restriction enzyme was chosen based on the following criteria: (1) no cutting of the expected breakpoint area; and (2) endonuclease activity would be unaffected by CpG methylation of the target sequence. A 100 ng aliquot of genomic DNA from both our patient and a control female was digested with the selected restriction enzyme in a total volume of 30 μl at 65 °C for 90 min. The reaction was inactivated using the QiaQuick PCR Purification Kit. A 20 μL sample of digested DNA was then mixed with 23 μL of DW, 5 μL of 10 × T4 ligase buffer (TaKaRa, Shiga, Japan) and 2 μL of T4 DNA ligase to make a final volume of 50 μL. Ligation reactions were incubated at 16 °C for 16 h. For subsequent PCR, 1 μL of digested and re-ligated DNA template was used in a total reaction volume of 25 μL with Tks Gflex DNA Polymerase (TaKaRa, Shiga, Japan). Primers were designed to avoid repetitive sequences (Additional file 1). The PCR conditions were as follows: 30 cycles of 10 s at 98 °C, 15 s at 60 °C, and 1 min at 68 °C. Amplified products were analyzed by gel electrophoresis and were purified following nested PCR (Additional file 1). The purified PCR products were sequenced via the standard Sanger method.

#### Breakpoint analysis on the other side

PCR was performed using Tks Gflex (TaKaRa, Shiga, Japan) to confirm the other side of the breakpoint sequence. Primer R which was previously designed for real-time PCR analysis of *OTC* upstream of exon 1 (i.e. *OTC* intron 1) was used as primer F in this reaction (Additional file 1). The PCR conditions and Sanger methodology were similar to those described above.

MLPA revealed the duplication of exons 1–6 of the *OTC* gene in our current study patient (Fig. 1a). We determined the range of the duplication using quantitative real-time PCR (Fig. 1b). We designed four qPCR experiments (U1-U4) between the promoter and enhancer regions to identify the upstream breakpoint. Likewise, we designed four qPCR assays (D1-D4) within intron 6 to identify the downstream breakpoint. In contrast to the male or female controls that showed ΔΔCt ratios of 0.5 or 1.0, respectively, the patient’s samples showed aΔΔCt ratio > 1.5 in some of these qPCR assays, suggesting that these regions were duplicated in this patient (Fig. 1c). The results indicated that the putative upstream breakpoints were located between PCR U3 and U4, and that the downstream breakpoints were between PCR D2 and D3.

**Fig. 2:**
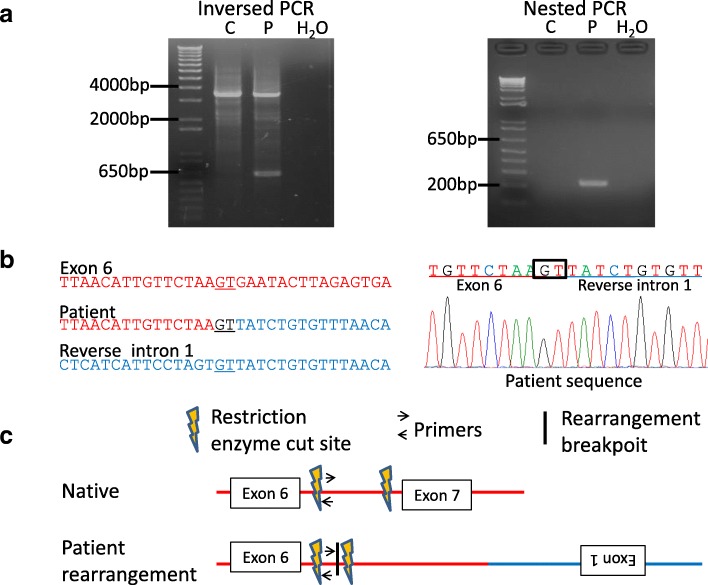
Identification of the duplication junction via inverse PCR. **a** Isolation of the junction fragment. Two distinct inverse PCR products were observed following agarose gel electrophoresis. The larger product was derived from a normal allele and the small product from a rearranged allele (left). The amplified products were purified following nested PCR (right). P, patient; C, control; H, H_2_O. **b** Sanger sequencing of the PCR products including the junction. The unknown sequence next to the junction was identified as intron 1 of the *OTC* gene in the reverse orientation. The normal exon 6 and intron 1 sequences are aligned in red and blue typeface, respectively. Underlined nucleotides indicate microhomology at the breakpoint junction. **c** Predicted structure of the junction. Horizontal arrows indicate the recognition sites of the primers used for inverse PCR and the vertical arrows denote the *Taq*I restriction sites

We next performed inverse PCR to analyze the genomic structure of the duplicated region. *Taq*I-digested DNA was used as a template to produce a 3.5 kb PCR product when amplified with inversely oriented intron 6 primers (Fig. 2a, c). However, an additional small PCR product was detected by agarose gel electrophoresis in the patient sample (Fig. 2a). The amplified products were sequenced after nested PCR (Fig. 2a). As expected, the breakpoint was located within intron 6 (Fig. 2b, c). Unexpectedly however, this breakpoint was found to be connected with intron 1 of the *OTC* gene in the reverse orientation*.* The breakpoint junction contained 2 nucleotides of microhomology at the fusion junction (Fig. 2b).

**Fig. 3:**
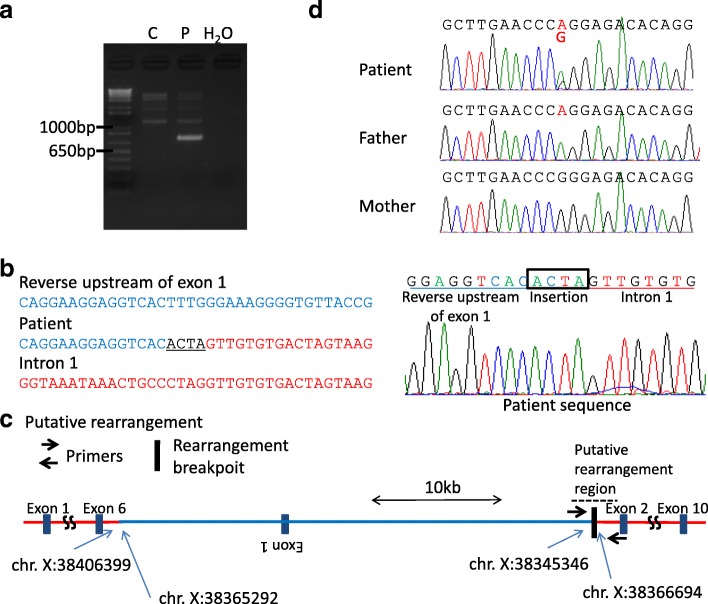
The complex rearrangement in the *OTC* gene of the study patient likely occurred via a replication-based mechanism. **a** Identification of the other junction by PCR. The PCR primer pair successfully amplified the junction product only from the OTCD study patient DNA. P, patient; C, control; H, H_2_O. **b** Sanger sequencing results for the PCR products including the junction. The normal sequence of the *OTC* gene upstream region and intron 1 are aligned in blue and red typeface, respectively. Underlined bases denote a non-templated microinsertion at the junction. **c** Predicted structure of the complex rearrangement leading to the *OTC* gene duplication. The positions of the PCR primers are indicated by black arrows. The first junction is indicated by a blue arrow. The nucleotide position of the breakpoints on the X-chromosome are also indicated. The position of the two breakpoints in intron 1 were found to be chr. X: 38365292 and chr. X: 38366694, which were 1402 bp apart. **d** Trio-genotyping of the common single nucleotide variant (rs752750694, NM 000531.5:c.-844C > T). The patient’s father carries A and her mother carries G/G. The patient was found to be an A/G heterozygote, but her A peak was two-fold higher than the G-peak. The areas under the curve (AUC) were 900 (A) and 328 (G) (Image J)

The other side breakpoint was analyzed using standard PCR with primers for the upstream breakpoint region and the breakpoint region in intron 1. The primer pair amplified only products from the patient’s DNA (Fig. 3a). By Sanger sequencing, the upstream region of the *OTC* gene was found to make an inverted connection with 1 (Fig. 3b, c). This breakpoint junction contained an additional 4 nucleotides (ACTA) of unknown origin (Fig. 3b). The positions of the two breakpoints in intron 1 were found to be chrX: 38365292 and chrX: 38366694, which were 1402 bp apart (Fig. 3c). We performed the same PCR amplification of both junctions in the patient’s parents but detected no products, suggesting that this complex rearrangement arose de novo. The patient’s duplicated region included a common single nucleotide variant (rs752750694, NM_000531.5:c.-844C > T). The patient’s father carries an A whereas the mother carries a G/G at this site (Fig. 3d). The patient was found to be an A/G heterozygote, but the peak of the A nucleotide was two-fold greater than the G-peak, suggesting that the patient carries two copies of A. These data suggest that the de novo duplication was of paternal origin.

## Discussion and conclusions

We here report the first documented case of OTCD caused by an exonic duplication of the *OTC* gene. Although the MLPA results for this case indicated a simple duplication of exons 1–6, further analysis indicated that it resulted from complex rearrangements. Two possible mechanisms have been proposed for such rearrangements: one is chromothripsis that is caused by chromosome shuttering followed by reunion, and the other is chromoanasynthesis that is a replication-based mechanism also known as fork stalling and template switching (FoSTeS)/microhomology-mediated break-induced replication (MMBIR). According to the replication-based model, the active replication fork can stall and switch templates using complementary template microhomology to anneal and prime DNA replication. This mechanism enables the joining or template-driven juxtaposition of different sequences from discrete genomic positions and can result in complex rearrangements [9].

In our current OTCD case, one junction presented 2 nucleotides of microhomology (GT), and the other junction manifested 4 nucleotides as a microinsertion (ACTA). Copy number variation with complex rearrangements and the presence of microhomology is indicative of a replication-based mechanism but the evidence for a non-templated microinsertion is noteworthy. Microinsertions are often observed in non-proofing DNA repair processes such as non-homologous end joining (NHEJ), which is activated by double-strand breaks [10]. However, a considerable body of evidence now suggests that microinsertions can be identified at junctions mediated by DNA replication-based mechanisms [11, 12]. A recent study has also suggested that an NHEJ-like pathway mediated by Polθ, which is an alternative NHEJ mechanism, may be induced by replication stress [13]. Taken together, an alternative NHEJ pathway might be activated during aberrant replication to restore DNA integrity, thus leading to chromoanasynthesis.

The evidence to date also suggests that de novo mutations occur more frequently in paternal alleles [14]. This bias is attributed to the higher number of DNA replication events in spermatogenesis than in oogenesis. Likewise, chromosomal structural variations are more frequently derived from the father [15]. The complex genomic rearrangements in our present patient were found to be of de novo origin but genotyping of a single nucleotide variant in the *OTC* gene demonstrated that the rearrangement allele originated from her father. Given the higher chance of the DNA replication errors during spermatogenesis, it might also reflect the replication-based mechanism.

MLPA can be used in the molecular diagnosis of several genetic diseases whose pathogenesis is related to the presence of deletions or duplications of specific genes [16]. Although deletions are clearly pathogenic, this is less certain in the case of duplications. In case of the *OTC* gene for example, duplications of the entire gene are innocuous and present as a normal variant in the general population [7]. In cases of partial duplication as seen in our current patient, gene function may not be necessarily be affected when the additional sequence is inserted into another genomic locus. Even in cases of a tandem duplication, it is feasible that one copy of the *OTC* gene may maintain an intact structure. In the current OTCD case, the inversion of exon 1 occurred together with its duplication. We predicted in this instance that this complex rearrangement would generate a tandem duplication of exons 2–6 and the production of truncated OTC proteins with defective function due to a frameshift or null protein expression due to nonsense-mediated mRNA decay. The functional defects caused by this mutant allele were therefore the cause of the OTCD in this woman.

In conclusion, we report the first case of OTCD caused by a complex rearrangement resulting in exonic duplication of the *OTC* gene. Our present report also emphasizes the necessity of fully investigating whether pathogenicity has resulted from a genomic duplication.

## Additional file

Additional file 1: PCR primers and genomic coordinates. (a) Primers for Sanger sequences of *OTC* exons. (b) Primers for qRT-PCR. (c) Other PCR. (XLSX 13 kb)

## Abbreviations

FoSTeS: Fork stalling and template switching
MLPA: Multiplex ligation-dependent probe amplification
MMBIR: Microhomology-mediated break-induced replication
NHEJ: Non-homologous end joining
OTC: Ornithine transcarbamylase
OTCD: Ornithine transcarbamylase deficiency
